# Salt and Aroma Compound Distributions Influence Flavour Release and Temporal Perception While Eating Hot-Served Flans

**DOI:** 10.3390/molecules26051300

**Published:** 2021-02-28

**Authors:** Marion Emorine, Chantal Septier, Christophe Martin, Sylvie Cordelle, Etienne Sémon, Thierry Thomas-Danguin, Christian Salles

**Affiliations:** 1 CSGA (Centre des Sciences du Goût et de l‘Alimentation), AgroSup Dijon, CNRS, INRAE, Université de Bourgogne Franche-Comté, F-21000 Dijon, France; marion_emorine@cotyinc.com (M.E.); chantal.septier@inrae.fr (C.S.); christophe.martin@inrae.fr (C.M.); sylvie.cordelle@inrae.fr (S.C.); etienne.semon@orange.fr (E.S.); thierry.thomas-danguin@inrae.fr (T.T.-D.); 2 Plateform ChemoSens, CSGA, F-21000 Dijon, France

**Keywords:** salt, aroma, flavour release, descriptive sensory analysis, temporal dominance of sensations, alternate time intensity, spatial distribution

## Abstract

To counteract the negative effect of salt overconsumption on health, strategies have been developed to reduce the salt content in food products. Among them, two promising strategies based on odour-induced saltiness enhancement and the heterogeneous distribution of flavour compounds were combined and assessed in four-layer cream-based snacks. To investigate the relationship between saltiness enhancement, temporal release and perception of flavour compounds in hot snacks with heterogeneous distribution of salt and aroma compounds, complementary techniques were used: nose space PTR-Tof-MS (Proton Transfer Reaction-Time of Flight–Mass Spectrometry) to assess the release of aroma compounds in vivo, and ATI (Alternate Time-Intensity) and TDS Temporal Dominance of Sensations) to evaluate perception as a function of time. The obtained results confirmed that the strategy of concentrating salt in the outer layer of a multilayer product was the optimal solution with respect to taste intensity. Heterogeneous salt distribution decreased aroma compound release and consequently aroma intensity but in different ways according to both salt and added aroma distribution in the food matrix. The salty taste enhancement could be due to the initial strong dominance of the salty sensation at the very beginning of the eating process. The involved mechanisms rely on a combination of physico-chemical and perceptual effects which are not clear yet.

## 1. Introduction

In the worldwide context of a healthy diet and in particular considering cardiovascular diseases, health agencies recommend products with lower salt contents to achieve better nutritional profiles [1,2,3,4]. In this way, several possible salt reduction interventions have been evaluated in many countries at different levels [5]. For example, millions of deaths could be prevented each year in the world if global salt consumption were reduced to the World Health Organization recommendation level of less than 5 g per day of salt intake for adults [3,6]. However, in many cases, low-salt food is not appreciated by consumers due to flavour drawbacks [7,8], although the appreciation level of salt-reduced food products varies according to the strategy and the type of food [9]. Thus, to avoid a negative economic impact, the reduction of salt content in processed foods without loss of consumer acceptability is a major issue for food manufacturers [10]. To reach this goal, various salt-reduction strategies have been proposed [11,12,13,14]. Among them, aroma–taste interactions and heterogeneous spatial distribution of tastants are promising strategies for taste enhancement.

The manipulation of the delivery of taste stimuli has been shown to enhance taste perception. As an example, salt perception in the mouth is enhanced by pulsatile stimulations [15]. This principle has been extended to layered gels alternating between low and rich domains of tastants. It has been shown that heterogeneous distributions of sugar [16], salt [17], fat [18], and odourants [19,20] increase perception intensity. Similar observations were reported in more complex foods for saltiness perception: bread [21], cream-based foods [22], and beef frankfurter [23], allowing a significant reduction in sodium content. Another promising way to enhance taste intensity is through cross-modal sensory interactions between aroma and taste. Food odours related to sweet foods (i.e., strawberry-like aroma) were found to enhance sweetness, while non-food-like odours were reported to suppress sweetness [24]. Concerning saltiness, it has been demonstrated that salt-associated odour can increase the salt perception of water solutions containing a low concentration of sodium chloride [25] and also in low-salt solid food matrices [26]. However, this effect is very dependent on the food matrix composition [27]. In cream-based food systems, a promising strategy combining olfactory-induced saltiness enhancement and heterogeneous distribution of stimuli was found to compensate for over a 35% decrease in salt content while maintaining saltiness intensity and consumer acceptability in hot snacks [22,28]. Only these authors reported a significant additive effect between the use of congruent aroma and heterogeneous distribution of salt for the enhancement of saltiness intensity, although the observed perceptual effect was limited because of salt and aroma compound diffusion between the different layers of the food. Surprisingly, no significant effect of aroma compound distribution on overall aroma intensity was observed. Furthermore, heterogeneous products were well liked by consumers compared to the homogeneous products [22,28]. In addition, in a composite food such as pizza, by modifying the salt content of each ingredient (ham, tomato, mozzarella, and dough), the overall salt content can be reduced by 30% while obtaining a salty perception higher than that of the control product [29]. These strategies, unlike the others that are proposed in the literature [30], allow an appreciable reduction in salt without or with a very limited amount of substituent or additive while ensuring good acceptability by the consumer.

Overall, the previous findings of [28] suggested that either saltiness in products with heterogeneous salt distribution counteracted aroma perception at a perceptual level or saltiness distribution modified aroma compound release because of the salting-out effect. Thus, we aimed to test the hypothesis that heterogeneous salt concentrations modify aroma compound release and further perception. Instrumental and sensory methods have been applied simultaneously to determine the effect of the spatial distribution of salt and aroma compounds in the food matrix on the dynamics of in vivo aroma compound release and perception over the time of consumption. Experiments were assessed by proton transfer reaction–time-of-flight–mass spectrometry (PTR-TOF-MS) and time-intensity and temporal dominance of sensations to follow the progressive release of salty and odourant stimuli and the dynamics of perception during the consumption of flans under the action of mastication and saliva.

## 2. Results

### 2.1. General Considerations

**Figure 1:**
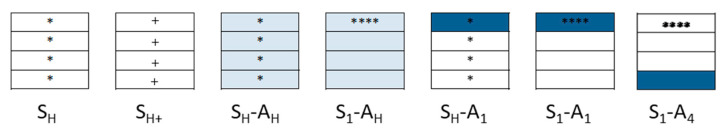
Overview of salt and added aroma distribution within the four layers in the four-layer cream-based snacks (FLPs). The symbol “*” represents the schematic initial salt distribution: “*” = homogeneous salt distribution (25% of overall added salt in each layer), “****” = heterogeneous salt distribution (100% of overall added salt located in one layer). S_H_+: unflavoured FLP containing 35% more salt than S_H_. Blue colours represent schematic initial added aroma distribution: “
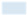
” = homogeneous added aroma distribution (25% of the overall added aroma mix in each layer), “
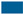
” = heterogeneous added aroma distribution (100% of the overall added aroma located in one layer).

Overall salt and volatile compound concentrations did not differ significantly between FLP (four-layer cream-based snacks) after heating, as previously reported for the same products, but diffusion of salt and volatile compounds was observed in each heterogeneous product. Indeed, for products with a heterogeneous distribution, after heating, 50% of the added salt and 40–60% of the added aroma (according to the nature of aroma compounds) were still present in the external layer. The rest migrated into the three other layers [22,28]. However, even if substantial diffusion was observed after the heating process, the initial design of salt and added aroma distribution was maintained, and products were found to still be sufficiently heterogeneous in comparison with homogeneous products (Figure 1). Moreover, these authors reported that salt and added aroma distribution had no effect on the rheological properties of the four-layer cream-based snack products.

### 2.2. Descriptive Analysis

**Figure 2:**
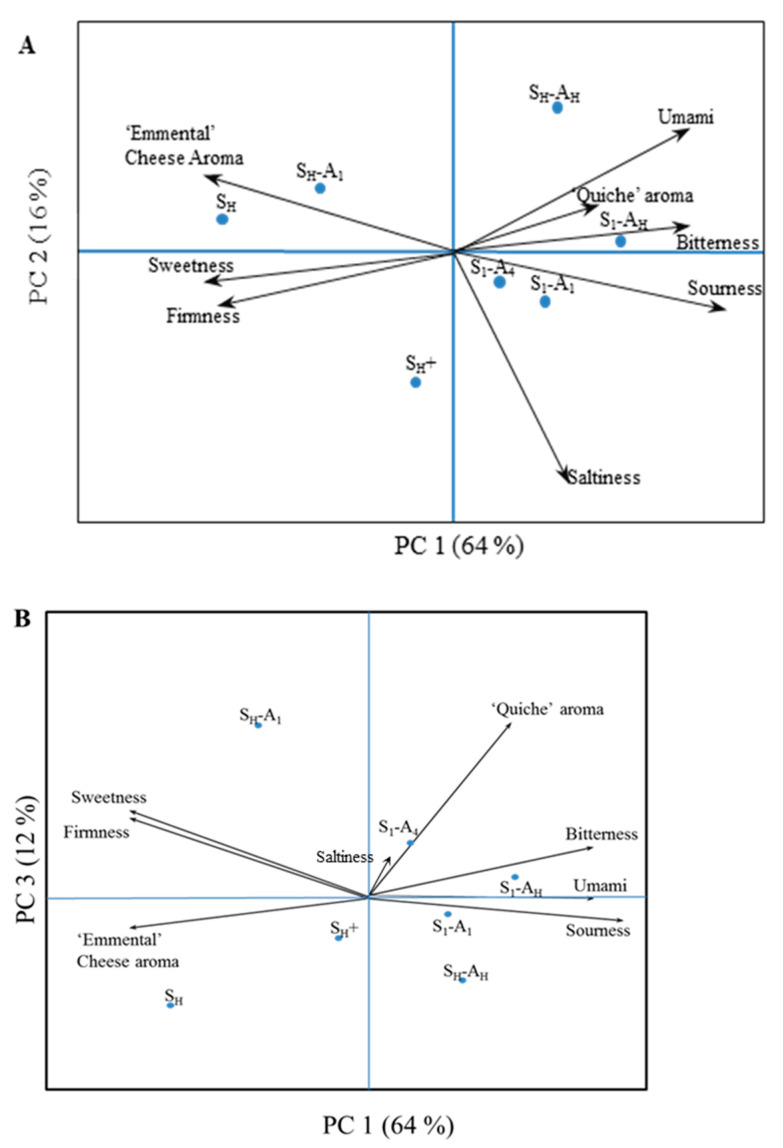
Principal component analysis (PCA) biplots (PC1,2 (**A**) and PC1,3 (**B**)) of the 8 attributes (taste, flavour, and texture attributes) rated during the descriptive analysis session for 7 four-layer cream-based products with 54 panellists. S_H_ and A_H_: homogeneous distribution of salt and aroma, respectively. S_n_ and A_n_: heterogeneous distribution of salt and aroma in layer n, respectively. S_H_+: contains 35% more salt than S_H_.

The configurations of the seven four-layer cream-based snacks (FLPs) are given in Figure 1. Principal component analysis (PCA) was performed to compare the seven products (Figure 2). The first component, accounting for 64% of the variance, was driven by all attributes except saltiness and “quiche” aroma. This component juxtaposes umami, sourness, and bitterness attributes (r < −0.87) with sweetness and firmness attributes (r > 0.84). The second component, accounting for 16% of the variance, was driven by the saltiness attribute (Figure 2A). The “quiche” aroma attribute did not contribute to the two first principal components; thus, a map representing the first and third components was drawn. The third component accounted for 12% of the variance and was driven by the “quiche” aroma attribute (Figure 2B).

Two-way ANOVAs (products as fixed factors and panellists as random factors) on intensity scores were performed for each attribute (Table 1). First, the results indicated a significant panellist effect for each attribute (F(53,330) > 4.3, *p* < 0.0001). Second, among the eight attributes, a significant product effect was found for saltiness, bitterness, “Emmental” cheese aroma, and “quiche” aroma. The value of the F ratio suggested that the FLP was best discriminated by the saltiness attribute.

**Figure 3:**
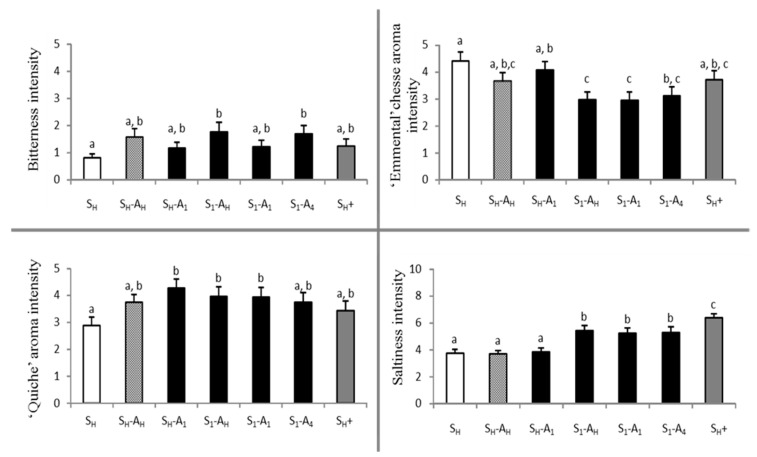
Mean intensity scores obtained for bitter, “Emmental” cheese aroma, “quiche” aroma, and salt attributes by descriptive analysis. Error bars represent the standard error of the mean. Identical letters indicate that odour intensity means are not significantly different at a level of 5% (Student–Newman–Keuls (SNK) test). S_H_ and A_H_: homogeneous distribution of salt and aroma, respectively. S_n_ and A_n_: heterogeneous distribution of salt and aroma in layer n, respectively. S_H_+: contains 35% more salt than S_H_.

Furthermore, post hoc tests were performed for the four attributes, which were found to significantly differentiate between samples (Figure 3). Significant differences between products were observed for bitterness intensity, which remained low for all samples. The reference (S_H_) was perceived as less bitter than two other samples (S_1_-A_H_ and S_1_-A_4_). In most cases, products with heterogeneous salt distribution (S_1_) were perceived as less intense in terms of “Emmental” cheese aroma compared to similar products with homogeneous salt distribution (S_H_). For the “quiche” aroma attribute, the post hoc test logically revealed an increase in aroma intensity for aromatised products compared to the non-aromatised reference (S_H_); however, all comparisons did not reach the 5% significance level. Interestingly, no significant difference was observed for “quiche” or “Emmental” aroma intensity as a function of added aroma distribution in either homogeneous or heterogeneous samples. Concerning the saltiness attribute, samples with a heterogeneous distribution of salt were found to be significantly saltier than samples with a homogeneous distribution, regardless of added aroma distribution (including the sample without added aroma). Furthermore, the saltiness reference (S_H_+), containing 35% more salt, was perceived as the saltiest product.

### 2.3. In Vivo Aroma Compound Release

The data concerning in vivo measurements performed on the FLP-containing odourants showed large variability between panellists for I*max*, t*max,* and AUC (Maximum intensity, time at which I*max* occurred, area under the curve, respectively), which are the representative parameters for both the amount of aroma compounds released and release kinetics (data not shown).

To investigate the effect of salt and added aroma distribution in layered products on aroma compound release, three-way ANOVAs (panellist as random factor, replication and product as fixed factors) were performed on each individual release kinetics parameter (I*max*, T*max*, AUC). The results of the three-way ANOVAs are presented in Table 2. For the two ions of interest (*m*/*z* 115.1117 and *m*/*z* 103.0754), a significant panellist effect was found (F(4,202) > 4.35, *p* < 0.001), whereas no significant replication effect was observed. Concerning the ion *m*/*z* 115.1117 (heptan-1-ol, related to the food matrix), a significant product effect on the t*max* parameter was found. A post hoc test indicated that t*max* was significantly shorter for the S_H_-A_H_ product than for the S_H_-A_1_ product. No significant product effect was observed for the two other extracted parameters. These results suggested that the kinetics of release for this volatile compound, originating from the FLP matrix and not from the added aroma, were mostly not affected by salt and added aroma distribution within the four layers.

**Figure 4:**
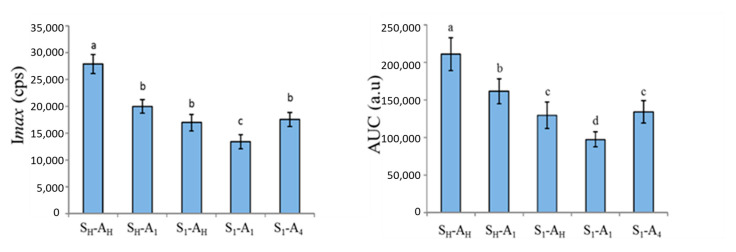
Effect of salt and added aroma distribution on two parameters (I*max*: maximum intensity and AUC: area under the curve) extracted from PTR-MS (Proton Transfer Reaction Mass Spectrometry) nose space curves for the peak at *m*/*z* = 103.0754 (ethyl propanoate). Values represent averages across assessors and replications. Error bars represent standard errors of the means. Identical letters indicate that the mean is not significantly different at a level of 5%. S_H_ and A_H_: homogeneous distribution of salt and aroma, respectively. S_n_ and A_n_: heterogeneous distribution of salt and aroma in layer n, respectively.

Concerning the ion *m*/*z* 103.0754 (ethyl propanoate, related to the added aroma), a significant product effect was found for I*max* and AUC but not for T*max*. A nonsignificant product effect for the T*max* parameter suggested that products had very similar texture properties and/or induced similar chewing behaviour. Post hoc tests were performed on I*max* and AUC parameters (Figure 4). They indicated that the maximum intensity (I*max*) was significantly higher for the homogeneous product (S_H_-A_H_) and significantly lower for the heterogeneous product containing added salt and added aroma in the upper layer (S_1_-A_1_) than for the three other samples (S_H_-A_1_, S_1_-A_H_, S_1_-A_4_; Figure 4). Concerning the area under the curve (AUC), while the results followed a similar trend as for I*max*, the differences were more pronounced (Figure 4). The two products with homogeneous salt distributions released significantly more ethyl propanoate than those with heterogeneous salt distributions.

Moreover, the product with a homogeneous distribution of both salt and added aroma (S_H_-A_H_) was found to have a larger AUC than the product with a homogenous distribution of salt but added aroma concentrated in only one layer (S_H_-A_1_). For products in which salt was concentrated in only one layer, it appeared that the product with salt and added aroma in the same layer (S_1_-A_1_) released less ethyl propanoate than the two other products with added aroma homogeneously distributed within the product (S_1_-A_H_) or concentrated in a single layer that was not the layer containing salt (S_1_-A_4_).

### 2.4. Time–Intensity Sensory Study

To investigate the effect of salt and added aroma distribution on salt and aroma perception over time of eating by the Alternate Time-Intensity method (ATI), the same parameters as for aroma compound release (I*max*, T*max*, and AUC) were extracted from the ATI curves obtained from each panellist, for each replicate, for each product, and for the two sensory attributes (saltiness and quiche aroma). Three-way ANOVAs (panellist as random factor, replication and product as fixed factors) were carried out on each extracted parameter for both sensations.

**Figure 5:**
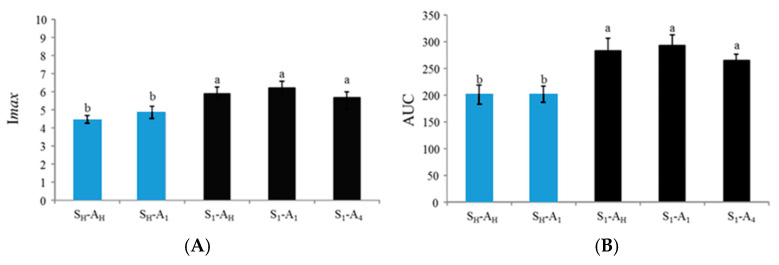
Mean Imax: maximum intensity (**A**) and AUC: area under the curve (**B**) for saltiness measured in FLP products using the alternate time–intensity method. Values represent averages across assessors and replications. Error bars represent standard error of the means. The same letters indicate that the mean is not significantly different at a level of 5%. S_H_ and A_H_: homogeneous distribution of salt and aroma, respectively. S_n_ and A_n_: heterogeneous distribution of salt and aroma in layer n, respectively.

Concerning salt perception, statistical analysis revealed a significant panellist effect (F(13, 189) > 5.71, *p* < 0.001) but no significant replication effect for the three parameters. These results confirm that each panellist had their own signature but that this signature was constant over replicates. A significant product effect was revealed for I*max* (F(4, 189) = 9.51, *p* < 0.001) and AUC (F(4, 189) = 9.44, *p* < 0.001) but not for T*max*. Post hoc tests indicated that, regardless of the added aroma distribution, products with heterogeneous salt distributions were perceived as significantly saltier than products with homogeneous salt distributions (Figure 5). These results were in line with those obtained by descriptive analysis and confirmed that heterogeneity of salt distribution, but not added aroma heterogeneity, induced higher salt perception compared to products with homogeneous salt distribution.

Concerning aroma perception, three-way ANOVA indicated a significant panellist effect (F(13, 189) > 8.21, *p* < 0.001) but no significant replication effect. Significant product effects were also revealed for I*max* (F(4, 189) = 4.32, *p* = 0.002) and AUC (F(4, 189) = 5.71, *p* < 0.001) but not for T*max*.

**Figure 6:**
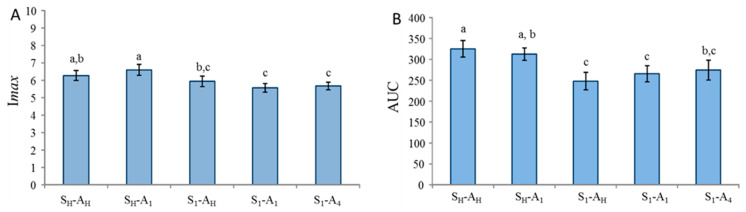
Mean I*max*: maximum intensity (**A**) and AUC: area under the curve (**B**) for the “quiche” aroma attribute. Every value represents the average over assessors and replications. Error bars represent standard error of the means. Identical letters indicate that the mean is not significantly different at a level of 5%. S_H_ and A_H_: homogeneous distribution of salt and aroma, respectively. S_n_ and A_n_: heterogeneous distribution of salt and aroma in layer n, respectively.

Post hoc tests carried out on I*max* indicated that the S_H_-A_1_ product was perceived with significantly higher maximal intensity than the three products with heterogeneous salt distributions (Figure 6). However, there was no difference between the two products with homogeneous salt distributions (S_H_-A_H_ and S_H_-A_1_) and no significant difference between the three products with heterogeneous salt distributions (S_1_-A_H_, S_1_-A_1_ and S_1_-A_4_). Post hoc tests carried out on the AUC parameter revealed a very similar trend in the two products with homogeneous salt distribution, inducing an AUC significantly larger than the three products with heterogeneous salt distribution, with the exception of S_1_-A_4_ compared to S_H_-A_1_.

Taken as a whole, the ATI results on aroma perception suggested that products with a homogeneous distribution of salt induce higher aroma intensity perception regardless of the added aroma distribution. These results were in accordance with the aroma compound release results, which indicated higher aroma compound release for S_H_-A_H_ and, to a lesser extent, S_H_-A_1_ products compared to the product with a heterogeneous salt distribution. However, the Pearson correlation between the release and perception data did not reach a significant level. Nevertheless, a significant correlation was found between AUC and I*max* for aroma compound release (r(5) = 0.99, *p* < 0.05) but not in the case of ATI results. Interestingly, correlations performed between ATI extracted parameters revealed significant negative correlations between AUC for aroma perception and I*max* but also AUC for salty intensity (r(5) = −0.94, *p* < 0.05; r(5) = −0.95, *p* < 0.05, respectively) and a significant negative correlation between I*max* for aroma intensity and Imax and AUC for saltiness ((r(5) = −0.98, *p* < 0.05, r(5) = −0.88, *p* < 0.05, respectively).

No difference was found for t*max*. This may be due to two factors. First, in our protocol, panellists were asked to rate aroma intensity at fixed times, and their previous rating was systematically recalled as a reference. This may have biased their judgement. Second, only three parameters were extracted from the curves, whereas other parameters, such as the initial slope or decrease slope, could provide relevant temporal information and may need further investigation.

Time to maximum intensity (T*max*) is an indicator of the rate of flavour release, which largely depends on the textural properties of food products and consequently on chewing conditions. In this study, although the chewing protocol was not imposed, the textural profile of the FLP was the same regardless of the salt and added aroma distribution, and no difference in aroma compound release was observed between products.

### 2.5. Temporal Dominance of Sensations

**Figure 7:**
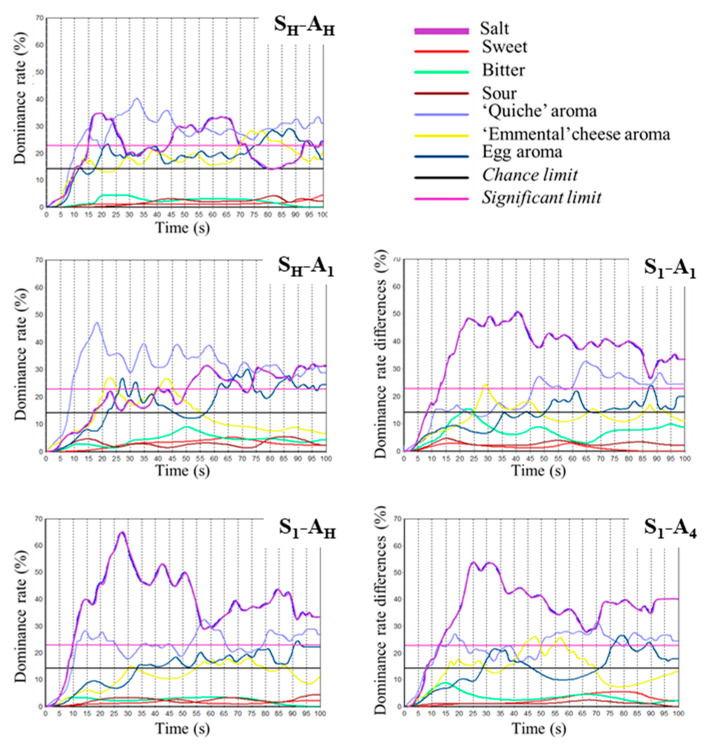
TDS (Temporal Dominance of Sensations) curves representing dominance rates in terms of subject percentages (15 panellists, 3 replicates) over a 100 s time period for consumption of products with homogeneous salt and added aroma distribution (S_H_-A_H_), heterogeneous added aroma distribution (S_H_-A_1_), heterogeneous salt distribution (S_1_-A_H_), heterogeneous salt and added aroma distribution (S_1_-A_1_ and S_1_-A_4_).

TDS (Temporal Dominance of Sensations) curves for FLP varying in salt and added aroma distribution between the four layers are presented in Figure 7.

Overall, the dominating sensations in the four-layer cream-based snacks were salty taste, “quiche” aroma, and, to a lesser extent, egg and “Emmental” cheese aroma. Nevertheless, the sequences of sensations differed according to stimuli distribution within the four layers.

The FLP with a homogeneous salt and compound aroma distribution (S_H_-A_H_) was characterised by a first significant short dominance of “quiche” aroma followed by a more pronounced dominance of salty taste); then, these two sensations were found to dominate alternatively. At the end of the eating sequence, all aroma sensations were found to be dominant. For the sample in which salt was homogeneous and aroma concentrated in one layer was added (S_H_-A_1_), the most dominant sensation was “quiche”, which was the only dominant sensation during the first half of the eating process. Then, salty taste dominated along with “quiche” aroma and egg aroma to a lesser extent. In the case of the sample with a heterogeneous salt distribution, regardless of added aroma distribution (S1), the dominance pattern was more or less the same, with a clear dominance of salty taste during the entire eating time and a significant dominance of “quiche aroma” in the second half of the eating process. Subsequently, the other aroma sensations also dominated in the second half of the eating process.

**Figure 8:**
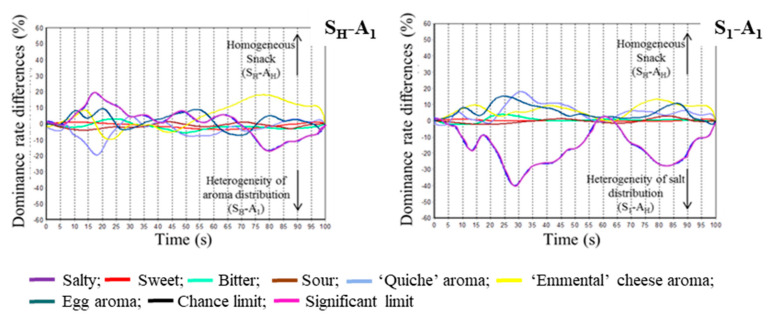
TDS (Temporal Dominance of Sensations) difference curves of products with homogeneous salt and added aroma distribution (S_H_-A_H_) and products with heterogeneous added aroma distribution (S_H_-A_1_) or heterogeneous salt distribution (S_1_-A_1_).

TDS difference curves highlighted that FLP differed mainly for the first dominant sensation, as well as for the last dominant sensation (Figure 8). TDS difference curves indicated that the product with homogeneous salt and added aroma distribution (S_H_-A_H_) was characterised by a more dominant salty taste at the beginning of the eating sequence, whereas the product with heterogeneous added aroma distribution (S_H_-A_1_) was characterised at the beginning of the eating sequence by a more dominant “quiche” aroma sensation. The contrast between these two products was also important at the end of the eating sequence. The “Emmental” cheese aroma dominates in the product with a homogeneous distribution of stimuli (S_H_-A_H_), while the salty taste dominates in the snack with a heterogeneous added aroma distribution (S_H_-A_1_). TDS difference curves also highlighted that salty taste was much more dominant in the product with a heterogeneous salt distribution (S_1_-A_H_) compared to the homogeneous reference (S_H_-A_H_), in which the aroma intensity sensations (“Emmental”, “quiche”, and egg descriptors) were comparatively dominant.

## 3. Discussion

The objective of the work was to investigate to what extent the heterogeneous salt distribution in a solid complex food matrix modifies aroma compound release and both aroma and salty taste perception.

The hypothesis of similar textures of the FLPs was supported by texture profile analysis results (not shown), which did not reveal any significant difference in their rheological behaviour. This meant that salt and added aroma distribution did not modify the textural properties of the products, which could in turn affect the overall oral process [31], the kinetics of flavour compound release [32,33], and the perceptual interactions between texture and flavour perception [34]. Thus, we can assume that variations in flavour compound release are mainly due to differences in their distribution in FLPs.

Among the FLP configurations examined in this study, differences in aroma compound release were observed, which can be explained in terms of interaction with the components of the food matrix and food oral processing. A rather similar effect of the salt and added aroma distribution was observed with nose space Imax and AUC. This can be explained by both the relative interdependency between these two temporal parameters and the overall same composition of each FLP configuration. In the case of S_H_-A_H_, salt and aroma were added homogeneously to the four layers; thus, surface/volume ratio partitioning in saliva was maximal for both stimuli, and the incorporation of salt and aroma compounds in saliva was maximal. If one considers that in this case, the surface of exchange with air in the mouth is maximal and drives release [35], this could explain the higher aroma compound release for this product. However, this interpretation does not hold true for the lower release of aroma compounds in the heterogeneous products. In that case, the results clearly demonstrated that salt distribution is involved in the aroma compound release process. An interpretation could be that mineral salts, including NaCl, have a release effect on aroma compounds [36]. Thus, the higher Imax and AUC observed in nose space are probably due to the salting-out effect [37,38], which is likely optimal in products with homogeneous salt distribution (S_H_-A_H_ and S_H_-A_1_). In summary, homogeneous distribution led to a higher surface exchange area between flavour compounds and saliva, which increased salt release and in turn salted out the effect in the S_H_-A_1_ product. Moreover, homogeneous distribution led to a higher surface exchange area between the product and in-mouth air, which increased volatile compound release, affording the greatest release from the S_H_-A_H_ product since both effects were combined. Following this reasoning, it seems logical that the lowest release was observed from the S_1_-A_1_ product, which is likely the least optimal in terms of the surface exchange area for aroma compounds but also for salt release and therefore for the salting-out effect.

The interpretation for the other products is less obvious. The S_1_-A_H_ product produced an intermediate level of release efficiency since it benefits from the surface exchange area for aroma compounds but has a lower salting-out effect. Concerning the S_1_-A_4_ product, the spatial distribution of the flavour compounds, as well as salt and aroma compound diffusion, was quite similar to S_1_-A_1_; however, salt and aroma were added to opposite layers of the product. To explain the higher aroma compound release observed for the S_1_-A_4_ product compared to S_1_-A_1_, one can consider the possible modifications in the extraction process of flavour compounds due to their different locations; in particular, S_1_-A_1_ contains three layers with a low amount of flavour compound stimulation, while S_1_-A_4_ contains only two layers with a low amount of flavour compounds. We can hypothesise that the change in flavour compound distribution leads to changes in their release kinetics, allowing a slower extraction of salt compared to aroma compounds. Differences in fat/salt interactions due to differences in flavour compound distribution could be an explanation, since lipids have already been shown to lower salt release in chewing conditions [39]. Moreover, the food matrix used in this study contained fat and proteins of different origins due to its complex composition. In such a matrix, salt concentration is known to influence the size of fat droplets [40,41,42]. One explanation is that Na^+^ binding led to a more hydrated and softer protein network, leading to bigger spaces available, and oppositely, the smaller size of the droplets in low-salt cheeses could explain an increase in mechanical resistance [43,44]. Thus, when the salt concentration is increasing, as for S_1_ compared to S_H_, the size of fat droplets is increasing, lowering their overall exchange surface area and thus lowering aroma compound release. This explanation is in line with the lower aroma release score of S_1_-A_H_, where only a quarter of aroma compounds have S1 salt concentrations, although the aroma release score is higher than the score of S_1_-A_1_. For S_1_-A_1_, all the aroma compounds have S_1_ salt concentrations, thus lowering the release of all the aroma compounds.

Another phenomenon that could be responsible for such differences is the possible effect of salt on salivary protein hydration, resulting in a higher quantity of aroma compounds in the head space for homogeneous salt distribution [45].

Concerning sensory perception, DA (Descriptive Analysis) and TDS were performed to obtain complementary information about the FLP sensory characteristics. The DA results are in accordance with previous studies [22,28] and confirmed that salty taste is perceived as more intense in products with heterogeneous distributions of salt. In line with previous studies, no effect of added aroma distribution on saltiness or on aroma intensity can be observed. However, more surprisingly and in contrast with previous studies, no effect of aroma addition on saltiness could be observed. This can be attributed to the addition of ethyl propanoate as a marker for flavour release. This aroma compound was added to ensure monitoring of the added aroma, but it induced a rather unpleasant aroma and bitterness, still perceptible within the food product despite the low quantity added.

TDS provided relevant additional information on temporal perception in heterogeneous food products. Indeed, similar TDS curves were obtained for all products with heterogeneous salt distributions. In these products, strong dominance of salt sensation was observed at the beginning of perception. Moreover, the salty taste was persistent at a significant dominance rate over the entire time of perception. In contrast, for products with a homogeneous distribution of salt, aroma sensation was found to be more dominant. Our hypothesis is that the high saltiness intensity could have prevented aroma from becoming dominant. Moreover, heterogeneity of salt distribution likely induced a “pulse” of salty taste at the very beginning of consumption. This first strong salt perception could explain the overall higher salty taste intensity observed in DA.

The snack products differed only in their distribution of flavour compounds within the four layers, no differences in texture attributes were logically observed in the DA evaluation, and TDS was performed only on the key taste and aroma attributes. Overall, the results of TDS are in line with the DA results, as previously reported. The authors of [46,47] demonstrated that TDS gave similar results to the ATI method, as well as the classic profile [48]. However, TDS revealed here that saltiness is likely the only sensation that captures panellist attention when eating products with a heterogeneous distribution of salt between layers, whereas homogeneous products tend to give a more complex pattern of sensations as a function of time, particularly a more balanced perception of aroma and salty taste highlighted by the alternance between aroma and salty taste dominance.

## 4. Materials and Methods

### 4.1. Four-Layer Cream-Based Snack Formulation

Seven variants of a four-layer cream-based snack (FLP) were developed. They were all composed of whipping cream, pasteurised eggs, Emmental cheese, modified food starch, wheat, salt, and mineral water. The FLPs were prepared according to the protocol described in [22]. The overall salt concentration was the same in each FLP: 5 g·Kg^−1^ table salt (Salin du Midi, Aigues-Mortes, France), except for one reference containing 35% more salt. An aroma mixture composed of ham aroma (30 g·Kg^−1^; Silesia, Gouvieux, France) and ethyl propionate (100 µL Kg^−1^; Sigma Aldrich, Saint-Quentin-Fallavier, France) was added to 5 FLPs. Moreover, the spatial distribution of salt and aroma varied from one layer to another according to the experimental design (Figure 1). The FLPs were coded according to the distribution of salt (S) and aroma (A). In indices, the letter _H_ refers to a homogeneous distribution of stimuli, and the numbers _1_ to _4_ refer to the spatial localisation of stimuli in the product; for example, S_1_-A_1_ refers to a product with salt and aroma mix added in the upper layer (see Figure 1).

### 4.2. Determination of Salt and Volatile Compound Concentrations

HPLC ionic chromatography (ICS Chain 3000, Dionex, Voisins le Bretonneux, France) was used to determine the overall salt content and salt content in the different layers after heating to measure salt diffusion between layers, according to [28]. The head space solid phase microextraction/gas chromatography/mass spectrometry (HS-SPME-GC-MS) method was used to determine the overall volatile compound concentration and to measure the diffusion of volatile components between the four layers after heating the FLP. The procedures are described in detail in [28].

### 4.3. Simultaneous Measurements of In Vivo Aroma Compound Release Kinetics and Temporal Perception

This study was conducted in accordance with the Declaration of Helsinki, and the protocol was approved by an ethical committee (Comité de Protection des Personnes Est-1, France, N° 2011/46) and by the French Agency for the Safety of the Healthcare products, AFSSAPS, France, N° 2011-A00807-34), and in accordance with the World Medical Association Helsinki Declaration as revised in October 2008. Fifteen panellists (10 women and 5 men, between 20 and 65 years old, average age 46 years) were recruited for the study. They were instructed not to smoke, eat, drink, or use any persistent perfume product for at least one hour before the session. Panellists gave their signed consent and received financial compensation for their participation. They were specifically trained to evaluate salt and aroma perception with single and complex solutions and to perform alternate time–intensity measurements in parallel with in vivo measurements.

#### 4.3.1. In Vivo Aroma Compound Release Monitoring

In vivo aroma compound release was measured using proton transfer reaction–time-of-flight–mass spectrometry (PTR-TOF-MS). Measurements were carried out using a commercially available PTR-TOF 8000 instrument (Ionicon Analytik GmbH, Innsbruck, Austria). Exhaled air from the nose was directly collected via a short plastic tube connected to the instrument via a heat peak line (temperature: 80 °C, internal diameter: 1 mm) at an average flow rate of 65.6 scans. Analytical parameters were set up during preliminary studies. During these pretests, ions corresponding to the specific fragments originating from volatile compounds contained in the sample were selected: [ethyl propanoate + H]^+^: *m*/*z* = 103.0754, which was linked to the aroma mixture, and [heptan-1-ol-H]^+^: *m*/*z* = 115.1177, which was linked to the food matrix. In addition to these two ions, [propane-2-one-H]^+^: *m*/*z* = 59.0491 was acquired to monitor the panellists’ breath.

The drift tube pressure was held at 2.4 mbar, and the overall drift voltage was 394 V. The measurements were performed at an E/N ratio of 90 Td (E: electric field strength in the drift tube; N: buffer gas number density in the drift tube; 1 Td = 10^−17^ V.cm^2^). The drift tube temperature was held at 80 °C. Analyses were performed with a scanning time of 108 ms. Spectra were recorded with a mass range from *m*/*z* 2.5 to 183.

Two sessions of 1.5 h were organised to obtain three replicates per assessor for each product. Each session started with an identical warm-up sample for all subjects (S_H_-A_H_ product), which was not included in the data analysis. This sample was used to train panellists to the task. The session continued with the consumption of 8 (for the first session) or 7 (for the second session) FLPs. The FLP samples (9.5 ± 0.3 g) were served at 50 °C in aluminium dishes. Samples were coded with three-digit random numbers and presented monadically and in a balanced order according to a William Latin square design. During each acquisition, several steps were recorded. First, acquisition of the ambient air for 150–200 scans to control the air in the sampling room was carried out, and then after positioning the inlet device in their nostril, panellists were asked to breathe normally to verify their exhaled air for 8 to 10 breaths. Finally, the exhaled air of the panellists during sample consumption was acquired until disappearance of the aroma signal. To do so, panellists were instructed to put the FLP at 50 °C in their mouth and to chew slowly and swallow it. Thus, on average, acquisitions lasted 4 min 40 s. During all measurements, panellists were asked to keep their mouths closed and to only breathe through the nosepiece. Between each sample, panellists were asked to rinse their mouth by eating apple wedges and drinking mineral water. All measurements were performed within a 2-week period.

For in vivo aroma compound release, parameters were extracted from each individual release curve: maximal intensities (I*max*), times at which I*max* occurred (t*max*), and areas under the curve (AUCs) [49]. The time at which products were placed in the mouth was used as the reference time for data comparison. Since our main objective was to compare aroma compound release between products, we used arbitrary units for release relative concentrations.

#### 4.3.2. Alternate Time–Intensity

The goal of this study was to investigate sensory differences in saltiness and aroma perception between products varying in the spatial distribution of salt and added aroma simultaneously with nose space measurements. Thus, a variant of classical TI methodology called alternative time–intensity (ATI) was used [50]. The ATI method allowed the measurement of changes in intensity over time of two key sensory attributes: salty taste and “quiche” aroma. Two training sessions were conducted to familiarise panellists with the methodology and to train them specifically to the key attribute alternate rating. Once the first attribute (the “quiche” aroma) appeared on the screen, they had 6 s to score their perception intensity on a 10 cm unstructured horizontal scale, labelled with the anchors “not” and “very” from left to right. After the first rating over a 6-s period, the length of rating was reduced to 4 s for all further ratings. Salt and “quiche” aroma attributes alternated over a period of 180 s.

Panellists were asked to rate attributes until the end of acquisition, even if they no longer perceived sensations. Furthermore, they were instructed to wait for the authorisation of the experimenter before disconnecting from the plastic tube connected to the PTR-MS. Between each sample, they cleansed their mouth with apple and water during a 3-min break. Sensory data acquisition was performed with FIZZ software (Biosystèmes, Couternon, France). Since the objective was to compare salty taste and “quiche” aroma perceptions between products, several variables were extracted from each sensory attribute and for each individual ATI curve: maximal intensities (I*max*), times at which I*max* occurred (t*max*), and area under the curve (AUC) [51].

### 4.4. Sensory Analysis Methods

Two different sensory methodologies were used: descriptive analysis (DA) and temporal dominance of sensations (TDS). The protocols were approved in the same way as reported in Section 4.3.

#### 4.4.1. General Common Conditions

Participants in both sensory studies had previous experience in descriptive sensory analysis. They declared to not suffer from food and other allergies and to not have any problem perceiving taste or smell. They were requested not to smoke, eat, or drink, except water, for one hour before the sessions. They did not receive any information about the aim of the experiment, but they signed an informed consent form for each test and were compensated for their participation (EUR 10 for a one-hour session).

Tests were conducted in an air-conditioned room (21°C) and under red light in individual booths. During the sensory sessions and before each tasting, the frozen food samples were heated in a vertical convection oven (Tecnox, Inoxtrend, Lucia Di Piave, Italy) for 4 min at 240 °C and served 1.5 min later at 55 °C. Products were served in a balanced order across subjects according to a Williams Latin square design. Each product was presented in an aluminium cup coded with random three-digit numbers.

Between each FLP, a 2 min break was imposed, during which the panellists were asked to rinse their mouth with apple and mineral water. A warm-up sample (S_H_-A_H_) was presented at the beginning of each session. Data generated from the warm-up samples were discarded from statistical analysis.

For both sensory methods, data were collected using FIZZ^®^ software (FIZZ Biosystèmes, Couternon, France).

#### 4.4.2. Descriptive Analysis (DA)

Fifty-four panellists (38 women and 16 men, mean age 44 years) took part in the DA study. DA was conducted to characterise the sensory profile of the seven FLPs. Panellists were asked to rate eight attributes: saltiness, sweetness, bitterness, sourness, umami, “Emmental” cheese aroma, “quiche” aroma, and firmness. The term “quiche” aroma was chosen following consensus between panellists. This attribute referred to the combination of the “ham aroma” [28] as well as to the smoked and chemical note induced by the addition of ethyl propanoate.

All attributes were presented simultaneously on the computer screen but in a random order, different for each panellist. Two samples of each FLP (2 × 9.5 g) were presented in each aluminium cup to provide a sufficient amount of product for the rating of the eight attributes. Panellists were instructed to eat the entire FLP products and to rate intensity for each attribute on a linear scale from “none” to “extremely strong”.

#### 4.4.3. Temporal Dominance of Sensation (TDS)

TDS was conducted to characterise the five aromatised FLPs: S_H_-A_H_, S_H_-A_1_, S_1_-A_H_, S_1_-A_1_, and S_1_-A_4_. Fifteen panellists (10 women and 5 men, mean age 46 years) were selected for the TDS study according to [28]. They were trained prior to the test sessions.

*Training sessions—*Seven attributes were chosen on the basis of results from a preliminary study (data not shown). The two-hour training session unfolded in three steps. First, subjects were trained in taste and flavour perception (saltiness, sweetness, bitterness, sourness, “quiche” aroma, “Emmental” cheese aroma, and egg aroma) using solutions containing flavourants or tastants at three different concentrations. Second, panellists were specifically trained on the TDS methodology with caramel custard containing sugar and caramel mixed at different concentrations. The dominance of sensations was explained to the subjects as a sensation that triggers their attention at that specific moment. Third, an FLP sample (S_H_-A_H_) was presented to panellists, who were asked to perform a TDS rating. This step was performed to familiarise panellists with the TDS procedure on the FLP using the target attributes.

*Test sessions—*All attributes were presented simultaneously on the computer screen. The order of attributes on the screen was randomised and differed between panellists [52]. One piece of product (9.5 g) was provided for each sample. Panellists were instructed to put the product in their mouth and simultaneously click on the “start” button. They were instructed to slowly eat the sample, and as soon as they perceived one sensation as dominant, they were asked to click on the corresponding attribute. When the dominant sensation changed, panellists chose the new attribute related to this dominant sensation until the perception ended. Panellists were free to select the same attribute several times and/or to never select an attribute as dominant. Moreover, it was made clear that an attribute was considered dominant until another attribute was selected. When panellists did not perceive any sensation, they were asked to click on the “end” button. A maximum time of 180 s was allocated for the evaluation of each sample. The data collected consisted of the attributes that were dominant and the corresponding time of their dominance. The evaluation of the four-layer cream-based products was performed in triplicate and took place in three sessions over a one-week period.

### 4.5. Data Analysis

#### 4.5.1. Time–Intensity

Statistical analyses of variance were carried out with in vivo release data and ATI data using the general linear model of STATISTICA^®^ Software (Version 10, StatSoft Inc., Maisons-Alfort, France). A Student–Newman–Keuls (SNK) post hoc test was also used to screen for significant differences between products. For all data analyses, effects were considered significant when *p* < 0.05.

#### 4.5.2. Descriptive Analysis

Data analysis was performed using STATISTICA^®^ software (version 10, StatSoft, France). Analyses of variance (ANOVA) and multivariate analysis of variance (MANOVA) were carried out with a general linear model (GLM). Student–Newman–Keuls post hoc tests were also performed. For all data analyses, the effects were considered significant when *p* < 0.05.

#### 4.5.3. Temporal Dominance of Sensations

TDS data were analysed with FIZZ software (FIZZ Biosystèmes, Couternon, France). For each product and each attribute, dominant rates were obtained over time by dividing the number of citations of that attribute (all replications) at any time by the number of panellists and the number of replications [47]. For each product and each attribute, the corresponding smooth TDS curves represented the percentage of panellists who selected the attribute as dominant at any one time, i.e., dominance rates. For the same products, TDS curves of all attributes were displayed on the same TDS graph. The higher the dominant rate, the better the agreement among panellists. Two additional X axes were added to the TDS graph to aid in interpretation. The chance level *Po* represents the dominance rate that an attribute obtains by chance (*Po* = 1/number of attributes). The significance level indicates that the smallest dominance rate was significantly higher than chance (*p* < 0.05).

TDS difference curves were also investigated to compare the two products. The curves are drawn by subtracting the dominance rates of each attribute at each evaluation time.

## 5. Conclusions

The results obtained in this study confirmed, through a temporal approach, that the heterogeneous distribution of salt can increase salty taste perception and thus could be a promising way to enhance salty taste intensity in reduced-salt food. Surprisingly, aroma compound release was not affected by aroma compound distribution but seemed to be driven only by salt distribution in the food product. Indeed, heterogeneous salt distribution decreased aroma compound release and consequently aroma intensity but differently according to both salt and added aroma distribution in the food matrix. Hypotheses on the mechanisms leading to these results rely on a combination of the physico-chemical effects of surface exchange area, salting out, changes in fat microstructure, and/or interactions with saliva proteins. These hypotheses should be confirmed by further specific research.

## Figures and Tables

**Table 1:** Mean intensity (standard deviation: SD), as well as panel discrimination among the 7 FLPs for each of the 8 attributes evaluated by descriptive analysis performed by 54 panellists. According to the F-ratio of the product factor ANOVA (Fisher statistics): *, **, and *** indicate a significant product effect at *p* < 0.05, *p* < 0.01, and *p* < 0.001, respectively, and NS indicates a nonsignificant product effect.

Attributes	Mean Intensity	SD	F
Bitterness	1.4	2.0	2.75 *
Sourness	1.5	2.1	1.7 NS
Sweetness	1.8	2.1	0.7 NS
Umami	2.1	2.3	1.8 NS
Saltiness	4.8	2.6	16.8 ***
Firmness	6.5	2.3	1.1 NS
Quiche	3.7	2.4	2.9 **
Emmental	3.6	2.5	4.6 ***

**Table 2:** Variables extracted from in vivo aroma compound release measurements (PTR-TOF-MS) obtained during the consumption of FLP for ions *m*/*z* 115.1117 (heptan-1-ol) and *m*/*z* 103.0754 (ethyl propanoate). Data were collected from each replication for each panellist. The T*max* values were calculated from the time at which the product was placed in the mouth. According to the F-ratio of the factor ANOVA: *, **, and *** indicate a significant product effect at *p* < 0.05, *p* < 0.01, and *p* < 0.001, respectively, and NS indicates a nonsignificant factor effect. AUC: area under the curve; Imax: maximum intensity; Tmax: time at which I*max* occurred.

Ions	Factor	Imax	Tmax	AUC
	Judge	15.71 ***	20.21 ***	115.54 ***
*m*/*z* 115.1117	Replication	1.59 NS	0.55 NS	0.29 NS
(heptan-1-ol)	Product	0.80 NS	2.50 *	0.18 NS
*m*/*z* 103.0754	Judge	8.55 ***	10.07 ***	31.16 ***
(ethyl propanoate)	Replication	2.71 NS	0.26 NS	0.50 NS
	Product	20.58 ***	1.69 NS	19.65 ***

## Data Availability

The data are not publicly available due to industrial confidentiality clauses.

## Sample Availability

Samples of the compounds are not available from the authors.
